# Comparison of muscle status and knee joint function between autologous hamstring tendon and peroneus longus tendon reconstruction of anterior cruciate ligament

**DOI:** 10.1097/MD.0000000000047342

**Published:** 2026-01-23

**Authors:** Xu Gao, Ji Fei, Yan Wang, Yuhai Yan, Wenwen Zhang, Kaiwei Zhang

**Affiliations:** a Guizhou University of Traditional Chinese Medicine, Guiyang, China; b Department of Orthopaedics, The First Affiliated Hospital of Guizhou University of Traditional Chinese Medicine, Guiyang, China.

**Keywords:** anterior cruciate ligament reconstruction, hamstring tendon, Myotonpro, peroneus longus tendon, retrospective analysis

## Abstract

This study aims to compare knee and ankle muscle/function outcomes following anterior cruciate ligament reconstruction using autologous hamstring tendon (HT) versus peroneus longus tendon (PLT). In this retrospective study, patients who underwent anterior cruciate ligament reconstruction with either PLT or HT grafts between 2015 and 2024 were analyzed. Postoperative follow-up data included muscle properties (tone-F, stiffness-S, elasticity-D) around the knee, International Knee Documentation Committee and Lysholm scores, and thigh circumference difference. For the PLT group, muscle properties (*F*, *S*, *D*) of ankle muscles (gastrocnemius, peroneus longus, tibialis anterior) and American Orthopedic Foot and Ankle Society scores were also assessed. Fifteen patients (average age 28 ± 9.83 years) were included. No significant differences were found between groups in knee muscle properties (*F*, *S*, *D*), International Knee Documentation Committee, or Lysholm scores (*P* > .05). However, thigh circumference reduction was significantly smaller in the PLT group (4.90 (4.00, 5.40)) compared to the HT group (3.45 (2.65, 4.33)) (*P* < .05). In the PLT group, ankle assessment revealed significant differences in elasticity (D) for the gastrocnemius and peroneus longus (*P* < .05) (gastrocnemius muscle: donor side [1.48 ± 0.04], healthy side [1.55 ± 0.02]; peroneus longus muscle: donor side [0.65 ± 0.27], healthy side [1.17 ± 0.03]), and in stiffness (*S*; donor side [391.60 ± 19.73], healthy side [433.60 ± 7.57]) and elasticity (*D*; donor side [1.07 ± 0.13], healthy side [1.41 ± 0.04]) for the tibialis anterior, with the healthy side showing superior values (*P* < .05). The American Orthopedic Foot and Ankle Society score was also significantly lower for the donor ankle side (92.20 ± 4.21) versus the healthy side (100.00 ± 0.00; *P* < .05). This study suggests that while both autologous PLT and HT grafts provide comparable postoperative knee function, the HT graft is associated with greater thigh muscle atrophy. The use of the PLT graft, however, leads to weakened muscle state and function around the donor ankle joint. Both grafts have distinct advantages and disadvantages, indicating that the choice should be tailored to the individual patient’s circumstances.

## 1. Introduction

The anterior cruciate ligament (ACL) plays an important role in maintaining the stability of the knee joint; injury to the ACL can destabilize the knee joint, causing joint instability, affecting the normal function of the knee joint movement, and causing osteoarthritis of the knee secondary to the injury.^[1]^ There are 50% of knee injuries related to ACL injuries, and most ACL injuries are non-contact.^[2,3]^ There are approximately 1,20,000 patients with ACL injuries in the United States each year; the incidence of ACL tears is 46/1,00,000 in Germany.^[4,5]^ Anterior cruciate ligament reconstruction (ACLR) is considered the “gold standard” for the treatment of ACL injuries. Bone-patellar tendon-bone has significant advantages in ACLR, such as bone-to-bone healing to accelerate healing of the graft, and complications such as increased pain in flexion of the knee, and a larger incision compared to popliteal tendon acquisition.^[6,7]^ The hamstring tendon (HT) is gradually gaining favor amongst surgeons because of its easier access and relatively few complications. The HT is a composite tendon tissue with the suture muscle originating from the anterior superior iliac spine and passing anteriorly through the thigh. It moves medially and inserts into the proximal tibia; the deep tendons of the thin femoral and semitendinosus muscles lie between the fascia of the foot and the superficial medial collateral ligament.^[8,9]^ The thin femoral and semitendinosus muscles are woven into 4-strand HT grafts as more commonly used grafts for arthroscopic ACLR.^[10]^ HT autografts are also confronted with complications after transplantation (donor site morbidity) such as knee pain as well as weakness in flexion and extension movements of the knee^[11]^; sensory deficit due to damage to the infrapatellar branch of the saphenous nerve. Between 39.7% and 88% of the patients experience this complication. The use of horizontal or oblique incisions reduces this risk.^[12]^

Currently, autologous peroneus longus tendon (PLT) is also being used concurrently in the clinic as a graft for ACLR. The PLT has a complex anatomy with a lengthy physiological configuration that can lead to calf, ankle, hindfoot and plantar symptoms. The proximal part of the peroneus longus muscle is located on the lateral aspect of the calf, and its distal tendon junction is located above the level of the ankle joint. The distal tendon of the peroneus longus has a long course with 2 sharp turns at the lateral ankle and hindfoot before inserting into the medial plantar.^[13]^ It has been demonstrated that the PLT is biologically strong enough to be used as a graft for ligament reconstruction^[14]^; resection of the anterior half of the PLT has no effect on foot morphology or gait parameters.^[15]^ In a 4-year ACLR follow-up study, it was found that using PLT as a graft did not affect ankle strength, and there was a difference in ankle dorsiflexion and extension activity at the donor site compared to the healthy side (*P* < .05), but this did not affect the use of PLT as a desirable material for ACLR grafts.^[16]^ A random controlled trial by Butt et al demonstrated that the 2 types of grafts, HT versus PLT, were not significantly different in the postoperative period after ACLR in terms of no significant difference in knee function, and the use of PLT grafts had no significant effect on the ankle joint.^[17]^ A meta-analysis showed that autologous PLT was superior to autologous HT in improving Lysholm and International Knee Documentation Committee (IKDC) scores (*P* < .05).^[18]^

In clinical practice, we have observed that autologous tendon grafts prepared using the PLT technique exhibit greater length and diameter. We hypothesize that autologous PLT may yield superior outcomes compared to autologous HT in cases of ACL rupture. Therefore, this retrospective study analyzed the differences between the 2 autografts in terms of functional recovery, complication rate and imaging performance after ACLR by comparing and analyzing them, with a view to providing some evidence-based basis for clinical graft selection.

## 2. Materials and methods

This study is a retrospective analysis study of patients with ACLR. Patients were identified with the diagnosis of ACL rupture and informed consent was obtained from patients for inclusion in this study. We conducted postoperative follow-up of patients who underwent ACLR between January 2015 and December 2024, all of whom had their surgeries completed in the Department of Orthopaedics of the First Affiliated Hospital of Guizhou University of Traditional Chinese Medicine (GUOTCM) with the same surgical team, and all of whom had their postoperative follow-up in the Orthopaedic Clinic of the First Affiliated Hospital of GUOTCM. Patient inclusion criteria were patients with isolated ACL rupture, aged 16 to 50 years old. Exclusion criteria were as follows: remaining associated ligament injuries, periprosthetic knee fractures, and the presence of pathological conditions in the lower limb or abnormalities of the contralateral knee. Functional scoring consisted of Myotonpro Muscle Conditioning Tester testing the *F* (muscle tone), *S* (muscle stiffness), and *D* (muscle elasticity) values of the muscles near the knee joint on the affected side (rectus femoris, medial femoris, lateral femoris, long head of the biceps femoris, semitendinosus, medial collateral ligament, and lateral collateral ligament) as well as providing the values of *F* (muscle tone), *S* (muscle stiffness), and *D* (muscle elasticity) of the muscles near the ankle joint on the PLT side (gastrocnemius, gastrocnemius longus, and tibialis anterior muscle). IKDC, Lysholm, thigh circumference (15 cm near the superior pole of the patella) discrepancy values (surgical side compared to the healthy side discrepancy values) were assessed using the American Orthopaedic Foot and Ankle Society (AOFAS) for the ankle donor site in the PLT group. This study was approved by the Ethics Committee of the First Affiliated Hospital of GUOTCM.

### 2.1. Surgical procedure

**Figure 1.**
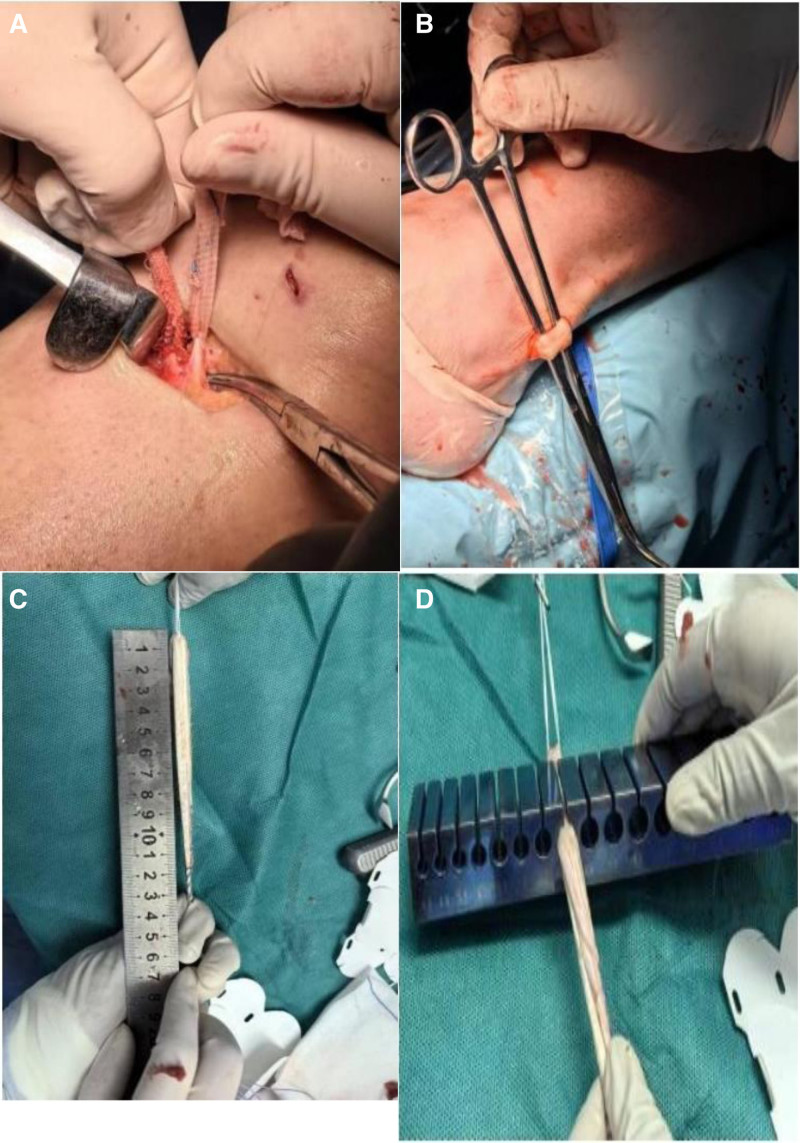
(A) Hamstring tendon. (B) Peroneus longus tendon. (C) Peroneus longus tendon. (D) Diameter measured after tendon fabrication.

The surgical team provided the patient with a detailed explanation of the characteristics of the 2 types of autologous tendons prior to the procedure, subsequently performing the tendon transfer according to the patient’s choice. The patient is placed supine under regional anesthesia and a tourniquet is applied to the thigh and inflated without elevation or bloodletting. Standard anterolateral and anteromedial portals were used. Diagnostic arthroscopy was performed to check for ACL rupture and then ipsilateral PLT or HT was collected. In the HT group, a 3 cm oblique skin incision was made on the hamstring at the proximal proximal anteromedial tibia. Both semitendinosus tendon and thin femoral tendon were harvested using an open tendon stripper. The tendons were then folded into 4-strand tendon grafts and secured at both ends using polyester sutures. For PLT, the location of the skin incision was marked, 2 to 3 cm above the ankle and 1 cm posterior to the lateral ankle. The incision was made through the skin, subcutaneous tissue, and superficial fascia. The peroneus longus and peroneus brevis tendons are identified. Tendon separation is performed 2 to 3 cm above the level of the lateral ankle. The PLT to the distal end of the short peroneal tendon is closed with an end-to-side suture. The peroneal long tendon is stripped proximally with a tendon stripper to approximately 4 to 5 cm above the fibular head to prevent peroneal nerve injury. Fibrous tissue was then removed from the intercondylar incision to facilitate visualization during tunnel preparation, but some of the remaining ACL fibers were retained as a reference for tunnel placement. The femoral and tibial tunnels were then prepared independently. After drilling, we proceeded to implant the tendon with buttons for graft fixation on the femoral side and with bioabsorbable screws for graft fixation on the tibial side after proper tensioning with a graft tensioner (Fig. 1A–D).

### 2.2. Postoperative follow-up assessment

Postoperative functional outcomes and donor site morbidity were documented postoperatively by direct patient examination and interview. A single orthopedic surgeon outside the surgical team examined and interviewed all patients. For patients who underwent ACLR with different grafts, we measured *F*, *S*, and *D* values of the muscles near the affected knee (rectus femoris, medial femoris, lateral femoris, long head of biceps femoris, semitendinosus, medial collateral ligament, and lateral collateral ligament) using the Myotonpro Muscle Conditioning Instrument and measured the IKDC, which was compared with the Lysholm scores and the difference in the patient’s and the healthy thigh circumference. For the PLT group, we also added measurements of *F*, *S*, and *D* values of the muscles near the ankle joint (gastrocnemius, peroneus longus, and tibialis anterior) as well as the AOFAS scale to assess the functional score of the ankle joint.^[12]^

### 2.3. Statistical analysis

The IBM SPSS Statistics 24.0 software (IBM Corp., Armonk) was used to analyze the data in this study, and for variables that meet the normal distribution, the mean + standard deviation was used; for skewed variables, the median (quartile) [*M*(*Q*25, *Q*75)] was used, and the independent samples *t*-test was used when dealing with data that meet the normal distribution; for skewed data, the Mann–Whitney *U* test. *P* < .05 was considered statistically significant. We performed a post hoc power analysis to confirm that our study had ≥80% power to detect the observed effect size. Calculations indicate a statistical power of approximately 15%. The data suggest that even if an effect exists, its magnitude may be very small (*d* = 0.2). Given this observed effect size, our study’s detection capability is quite limited. This negative result may indicate that the effect does not exist, or that it is too small to be detected without an extremely large sample size.

## 3. Results

### 3.1. Demographic characteristics

This retrospective observational analysis of the clinical efficacy of ACLR included a total of 15 patients. Autologous HT was used in 10 patients, and autologous PLT in 5 patients. There were 11 males and 4 females. The shortest follow-up duration was 1 month, the longest was 68 months, with an average follow-up duration of 11.75 months. The youngest patient was 16 years old, and the oldest was 48 years old. Seven patients sustained injuries due to sports-related activities, and 8 patients sustained injuries due to non-sports-related activities. All patients’ basic demographic characteristics are shown in Table 1.

**Table 1:** Patient characteristics.

Group	Gender	Age	BMI	Types of injuries	The injured side	Time to last follow-up after surgery (mo)
HT group	M: 7	26.70 ± 9.21	23. 15 ± 2.85	Sporting: 4	L: 6	15.1 ± 22.34
	F: 3			Non-sporting: 6	R: 4	
PLT group	M: 4	30.60 ± 11.59	26.25 ± 7.17	Sporting: 3	L: 4	8.40 ± 3.85
	F: 1			Non-sporting: 2	R: 1	

BMI = body mass index, HT = hamstring tendon, PLT = peroneus longus tendon.

### 3.2. F (muscle tone), S (muscle stiffness), D (muscle elasticity)

In the present study using autologous PLT versus autologous HT to perform ACLR in both groups of patients, the values of *F*, *S*, and *D* of the muscles near the knee joint on the affected side (rectus femoris, medial femoris, lateral femoris, biceps femoris longissimus, semitendinosus, medial collateral ligament, and lateral collateral ligament) were measured by the Myotonpro Muscle Condition Detector, and there was no significant difference between both groups in terms of the *F*, *S*, and *D* of the above muscles were not significantly different (*P* > .05; Table 2).

**Table 2:** *F*, *S*, and *D* of the periprosthetic muscles or ligament of the knee joint in the HT group versus the PLT group.

	*F*		*S*		*D*	
Muscles or ligaments	PLT group	HT group	PLT group	HT group	PLT group	HT group
Rectus femoris	11.80 (11.45, 12.45)	11.95 (11.43, 12.48)	214.00 (206.00, 219.00)	223.00 (212.00, 236.75)	1.77 (1.66, 1.82)	1.81 (1.69, 1.83)
Medial femoral muscle	12.70 (11.95, 13. 10)	12.80 (11.95, 13.30)	235.00 (231.50, 238.00)	238.00 (218.25, 242.25)	1.56 (1.55, 1.64)	1.60 (1.54, 1.70)
Lateral femoral muscle	16.70 (15.85, 17.85)	16.35 (15.55, 18. 15)	341.00 (331.50, 356.00)	347.50 (323.50, 357.00)	1.51 (1.47, 1.53)	1.52 (1.40, 1.59)
Long head of the biceps femoris	12.00 (11.60, 12.25)	11.85 (11.40, 12. 13)	201.00 (197.50, 207.50)	205.00 (158.75, 214.00)	1.95 (1.83, 2.01)	1.79 (1.36, 2.01)
Semitendinosus	13.00 (12.15, 13.05)	13.20 (12.80, 13.53)	233.00 (232.50, 236.50)	238.00 (229.25, 244.25)	1.65 (1.57, 1.66)	1.59 (1.41, 1.66)
Medial collateral ligament	15. 10 (14.05, 15.30)	15. 15 (14.83, 15.20)	377.00 (359.00, 385.50)	360.00 (333.00, 373.00)	1. 19 (1.13, 1.22)	1.21 (1.18, 1.25)
Lateral collateral ligament	21.30 (19.00, 26.40)	20.80 (19.28, 23.33)	987.00 (793.50, 1049.00)	844.50 (747.25, 911.25)	1. 16 (1. 11, 1.22)	1.21 (1.17, 1.31)

HT = hamstring tendon, PLT = peroneus longus tendon.

In the present study, the *F*, *S*, and *D* values of the nearby muscles (gastrocnemius, peroneus longus, and tibialis anterior) of the ankle joint provided with autologous PLT grafts were measured and compared with the healthy side. The *F* and *S* values of gastrocnemius and peroneus longus were not significantly different from the healthy side (*P* > .05), and the *F* values of tibialis anterior were also not different from the healthy side (*P* > .05). In contrast, the *S* (donor side [391.60 ± 19.73], healthy side [433.60 ± 7.57]) and *D* (donor side [1.07 ± 0.13], healthy side [1.41 ± 0.04]) values of the tibialis anterior muscle were significantly different in the comparison between the 2 groups (*P* < .05), and the *D* values of the gastrocnemius (donor side [1.48 ± 0.04], healthy side [1.55 ± 0.02]) and peroneus longus muscles (donor side [0.65 ± 0.27], healthy side [1.17 ± 0.03]) were also different (*P* < .05), and all of them were better in the control group (HT group) than in the experimental group (PLT group; Table 3).

**Table 3:** *F*, *S*, *D* of the muscles around the ankle joint on the donor side and the healthy side in the PLT group.

					(Mean ± SD)	
	*F*		*S*		*D*	
Muscle	PLT group	HT group	PLT group	HT group	PLT group	HT group
Gastrocnemius muscle	12.22 ± 1. 11	13.74 ± 1.05	228.20 ± 11.61	231.40 ± 6. 11	1.48 ± 0.04	1.55 ± 0.02
	*P* > .05		*P* > .05		*P* < .05	
Peroneus longus	15.46 ± 1.50	16.76 ± 0.76	341.20 ± 19.15	330.00 ± 19.62	0.65 ± 0.27	1. 17 ± 0.03
	*P* > .05		*P* > .05		*P* < .05	
Tibialis anterior muscle	18. 10 ± 1.01	18.42 ± 0.53	391.60 ± 19.73	433.60 ± 7.57	1.07 ± 0.13	1.41 ± 0.04
	*P* > .05		*P* < .05		*P* < .05	

HT = hamstring tendon, PLT = peroneus longus tendon, SD = standard deviation.

### 3.3. IKDC, Lysholm, thigh leg circumference difference (difference value between healthy and affected side)

Comparison of knee function between PLT group and HT group by IKDC, Lysholm revealed that there was no significant difference between the 2 groups (*P* > .05). There was a statistically significant difference between the 2 groups in terms of thigh leg circumference difference (*P* < .05), with the experimental group (4.90 [4.00, 5.40]; PLT group) having more changes in thigh leg circumference than the control group (3.45 (2.65, 4.33); HT group; Table 4).

**Table 4:** IKDC, lysholm and changes in thigh leg circumference in HT and PLT groups.

			*M*(*Q*25, *Q*75)
	PLT group	HT group	*P* value
IKDC	77.01 (74. 14, 79.89)	74.71 (73.56, 77.59)	05
Lysholm	89.00 (85.00, 95.00)	90.00 (85.50 , 90.00)	.05
Change in thigh circumference	4.90 (4.00, 5.40)	3.45 (2.65, 4.33)	.05

HT = hamstring tendon, IKDC = International Knee Documentation Committee, PLT = peroneus longus tendon.

### 3.4. American Orthopedic Foot and Ankle Society

Comparing the AOFAS of the ankle on the side of PLT provided (both ACL injured side) with the healthy side, there was a significant difference between the 2 groups (*P* < .05), and the control group (100.00 ± 0.00; healthy side) was better than the experimental group (92.20 ± 4.21; donor side; Table 5).

**Table 5:** Donor-side versus healthy-side AOFAS in PLT group.

			(Mean ± SD)
	PLT group	HT group	*P* value
AOFAS	92.20 ± 4.21	100.00 ± 0.00	.05

AOFAS = American Orthopedic Foot and Ankle Society, HT = hamstring tendon, PLT = peroneus longus tendon, SD = standard deviation.

## 4. Limitations

This study has certain limitations. As it is a retrospective, single-center design, the patient sample analyzed was relatively small. Furthermore, our analysis focused solely on the relationship between graft type and muscle status post-ACLR surgery and knee function, without fully accounting for patient-related factors such as socio-occupational status or exercise habits. Other potential confounding factors were not fully accounted for, including variations in the time span between initial surgery and final follow-up, differing stages of postoperative recovery among patients, and irregular outpatient follow-up schedules. This precludes assurance regarding patients’ adherence to and completion of postoperative rehabilitation programmes. Future research could be enhanced through multicentre designs and randomized controlled trials. And the Myotonpro can only detect superficial muscles or tendons, and cannot detect deep muscles.

## 5. Discussions

In this retrospective study, we used the Myotonpro Muscle Conditioning Test to examine *F*, *S*, and *D* in the periprosthetic muscle groups of the knee and some muscle groups of the ankle joint in patients who underwent ACLR using 2 different autologous tendons (PLT or HT), and we evaluated the postoperative function of the knee and ankle in the subjects. It indicates that there is no difference in the effect of the 2 autologous tendon grafts on the muscles around the knee joint after ACLR, but the use of autologous PLT will have a certain effect on the muscle elasticity of the gastrocnemius and peroneus longus muscles of the ankle joint on the donor side and on the muscle hardness and muscle elasticity of the tibialis anterior muscle.

Both quadriceps and hamstring strength after ACLR surgery using autologous HT decreases in the early postoperative period and then gradually regains its strength.^[19]^ The use of autologous PLT in ACLR also causes a decrease in the strength of the rectus femoris, intermediate femoris, lateral femoris and medial femoris muscles in the postoperative period.^[20]^ Strength power of the quadriceps is essential for the maintenance of normal knee function, and an important potential factor in quadriceps weakness after knee trauma is the possible consideration of Arthrogenic muscle inhibition (AMI),^[21]^ AMI being a reflex response that persists after joint injury. It describes the inability to fully contract a muscle despite the lack of structural damage or innervation. It is considered a reflex response to joint injury because it is beyond conscious, voluntary control.^[22]^ AMI results in decreased muscle activation, which impairs muscle strength and leads to abnormal movement biomechanics. AMI is often resistant to traditional rehabilitation techniques, which leads to persistent neuromuscular deficits after ACLR.^[23]^ Impairment of quadriceps strength after ACLR is common and associated with altered knee biomechanics.^[24,25]^ Reduced quadriceps strength is associated with negative short- or long-term outcomes such as low return to sport rates, reduced quality of life and early onset osteoarthritis.^[26-28]^ Peripheral changes (morphological and cellular) in the quadriceps after ACL injury or reconstruction can lead to long-term weakness of the quadriceps.^[29]^ Other cellular adaptations in the lateral femoral muscle due to ACL injury include increased extracellular matrix and decreased satellite cells prior to surgery.^[30]^ The result of a larger muscle extracellular matrix is that the active contractile component occupies less area, effectively reducing force production. In addition, reduced satellite cell content in injured muscles may impair the muscle’s ability to respond to subsequent rehabilitation. Satellite cell content is strongly associated with strength gains following resistance training in older adults, with satellite cells providing myonuclei by fusing into growing fibers.^[31]^ Traditional physiotherapy techniques have had limited success in attenuating alterations in quadriceps composition. However, blood flow restriction training has been suggested as a potential method to improve these deficits.^[32]^

Postoperative hamstring strength was lower after reconstruction using HT grafts, and weakness of the knee flexors was present for 2 years postoperatively but was not observed again after 2 years.^[33]^ The semitendinosus and thin femoral tendons regenerated and returned to almost normal goosefoot stops for at least 6 years after ACLR acquisition. The cross-sectional area of the regenerated tendons was similar to that of the unoperated contralateral tendon. The patient had a strength deficit in deep knee flexion but not in internal knee rotation.^[34]^ Among the rest of the studies, patients with autologous HT experienced a deficit in knee flexion strength postoperatively, although it may return to the same level as the healthy side after a few years,^[35,36]^ and other grafts causing fewer postoperative complications or more comprehensive postoperative rehabilitation may need to be considered to address this deficit. Postoperatively, attention may be directed towards neural control and muscular performance, both of which can enhance hamstring strength and proprioception, this may reduce bilateral kinematic discrepancies and improve landing control following ACLR surgery.^[37]^

Muscles near the ankle joint were considered to be associated with foot morphology and gait through the ankle joint, whilst maintaining ankle joint stability.^[38,39]^ And the study by Zhang et al used PLT for ACLR to achieve satisfactory knee function. However, it does affect donor ankle inversion and eversion and therefore requires postoperative exercise. Despite the increased inversion-eversion motion in the operated foot, subjective function scores were similar in the operated and non-operated foot, which may be influenced by the subjectivity and margin of error of the AOFAS scores and the relatively small variations in ankle inversion-eversion angles.^[40]^ The use of PLT grafts in another study did not significantly affect ankle function.^[41]^

Changes in muscle length can affect muscle endurance, so the endurance of the peroneus longus muscle may change after autologous PLT is removed.^[42]^ The peroneus longus muscle has a complex anatomical structure and a relatively long length, which can lead to symptoms involving the lower leg, ankle joint, hindfoot, and plantar region. Proximally, the peroneus longus muscle is located in the lateral compartment of the lower leg, with its distal tendon insertion above the ankle. Pathological changes in the peroneus longus muscle not only affect the ankle joint but may also impact the knee joint.^[13,43]^ Patients with chronic ankle instability exhibit increased muscle tension and stiffness in the peroneus longus and tibialis anterior muscles, accompanied by reduced elasticity.^[44]^ Based on the findings in this study, patients using PLT exhibit chronic ankle instability on the donor side of the ankle joint, and patients with chronic ankle instability have the characteristic of static muscle stiffness in the ankle joint plantar flexor muscles.^[45]^ The gastrocnemius muscle has long been regarded as an important component of the knee joint, playing a role in knee flexion and stability at all knee and ankle joint angles. The gastrocnemius muscle does exhibit age-related changes in mechanical properties, including reduced tensile strength, tensile stress, and elastic modulus, which are associated with collagen fiber deformation and excessive fat accumulation at the muscle-tendon junction. These changes may further lead to decreased muscle strength and mass with aging, particularly in individuals over 60 years of age.^[46]^ The medial and lateral heads of the gastrocnemius muscle not only contribute to dynamic stability of the ankle joint but also facilitate plantar flexion and forward movement of the ankle. In these functions, the muscle’s elastic properties directly influence contraction speed and force generation. Regulation of muscle elastic properties can reflect pathological changes and recovery effects.^[47]^ Increased gastrocnemius muscle tension causes pain and simultaneously affects ankle joint function; when gastrocnemius muscle tension exceeds 13°, it is considered abnormal. Most patients with foot and ankle disorders do not exhibit abnormal gastrocnemius muscle tension, but over one-third of patients with forefoot disorders have gastrocnemius muscle tension.^[48,49]^ Changes in ankle joint function or surrounding muscle tissue after autologous platelet-rich plasma therapy are not solely due to tendon deficiency. Following ACLR, alterations in lower limb biomechanics, joint angles, and torque also influence ankle joint function. Fontenay et al compared dynamic analyses of the affected and unaffected lower limbs post-ACLR, finding that the affected side exhibited reduced total torque and total power compared to the unaffected side. There were no differences in maximum hip and knee joint strength between the 2 legs. The maximum ankle joint strength was 34% lower on the affected side than on the unaffected side, and the ankle joint plantar flexion angular velocity was 31% lower. Post-ACLR, the affected limb exhibited changes in movement.^[50]^

There was no significant difference in IKDC scores and Lysholm knee scores between the popliteus and PLT groups at 6 months and 1 year after surgery in 194 patients with ACLR. The mean AOFAS scores of the PLT and HT groups were not different from those in the AOFAS group. AOFAS mean scores were not different. Similar knee stability and functional outcomes were observed in both groups and there was no significant donor site morbidity between the 2 groups.^[51]^ The final tensile strength of double-stranded PLT and quadruple-stranded HT was significantly higher than that of natural ACL in the study by Shi et al The final tensile strength of double-stranded PLT was comparable to that of quadruple-stranded HT. There were no significant differences in clinical or functional scores between the 2 groups. There were no significant differences in preoperative and postoperative biomechanical testing of the donor ankle joints. PLT is an alternative autograft suitable for ACLR without significant biomechanical disadvantages to the ankle donor site.^[52]^ He et al demonstrated that PLT autografts had similar functional outcomes and graft survival in ACLR by comparing them with HT autografts. However, a slight decrease in AOFAS score should be considered during surgical planning. Therefore, PLT is a suitable extra-articular knee autograft for ACLR to avoid complications of quadriceps-popliteus imbalance that may occur during knee autograft transplantation.^[18]^

Within the range of <7.0 to >9.0 mm, the risk of failure correspondingly decreases for every 0.5 mm increase in graft diameter. Failure is multifactorial; however, maximizing graft diameter to match each patient’s anatomical space without overfilling represents an effective preventive measure surgeons can adopt to reduce failure.^[53]^ Smaller graft diameters correlate with poorer knee injury and osteoarthritis prognosis scores, reduced recreational function scores, and increased revision surgery rates. PLT exhibits a marginally larger diameter than HT (8.63 mm vs 8.08 mm), and its biomechanical properties resemble those of the native ACL, potentially contributing to its durability.^[18]^ From a biomechanical perspective, the strength of PLT repair materials is comparable to or exceeds that of native ACL and HT repair materials. Studies report that the ultimate tensile strength of both double-strand PLT and quadruple-strand HT significantly surpasses that of the native ACL (*P* < .05), while the in vitro ultimate tensile strength of double-strand PLT is equivalent to that of quadruple-strand HT. This robustly demonstrates that PLT possesses tensile strength comparable to the natural ACL, further underscoring the importance of prioritizing PLT graft materials in ACL reconstruction.^[52]^ Consequently, as ACLR grafts, PLT exhibits distinct advantages over HT, namely a larger graft diameter and superior biomechanical strength.

**Figure 2.**
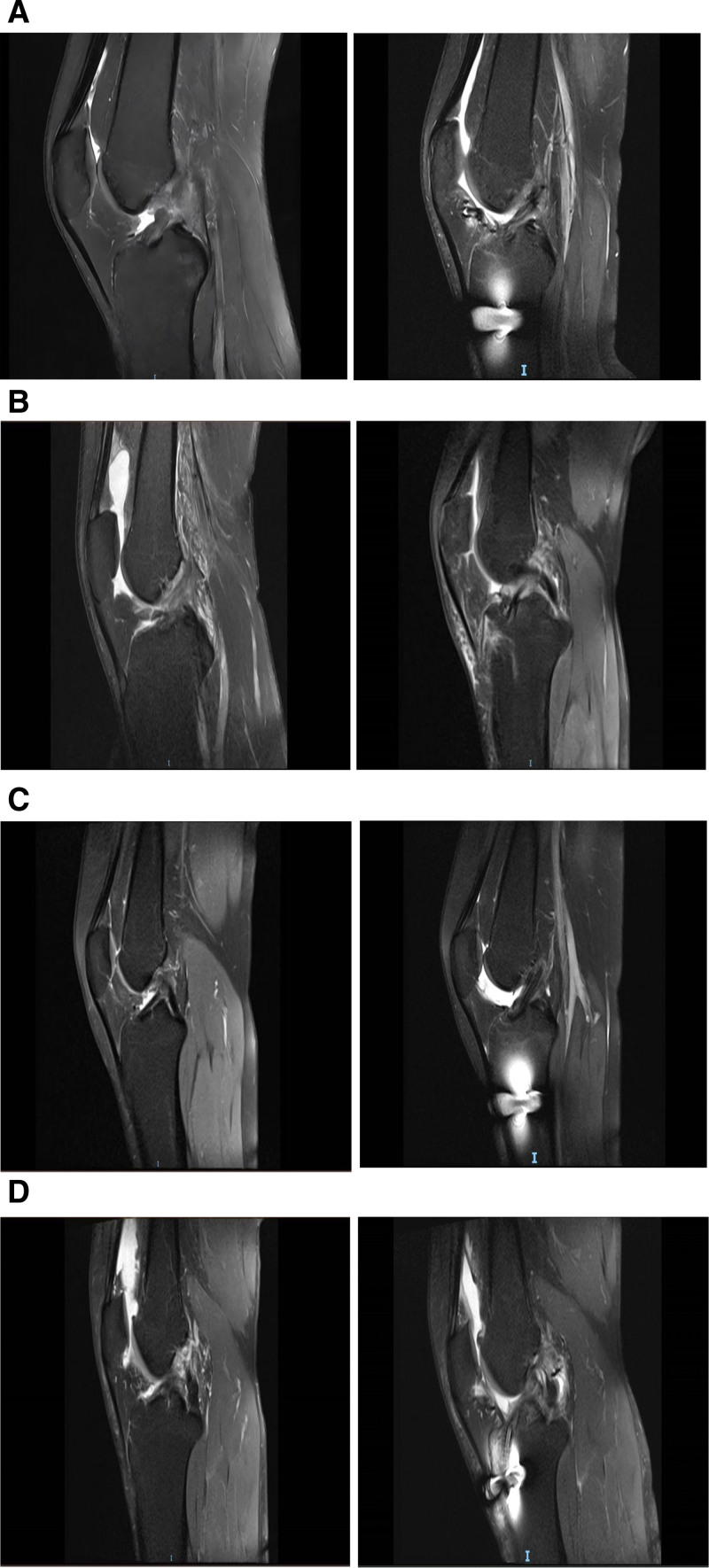
Figure (A1) shows the preoperative magnetic resonance imaging result of patient no. 1 in the PLT group. (A2) Figure shows the postoperative magnetic resonance imaging result of patient no. 1 in the PLT group. (B1) Figure shows the preoperative magnetic resonance imaging result of patient no. 2 in the PLT group. (B2) Figure shows the postoperative magnetic resonance imaging result of patient no. 2 in the PLT group. (C1) Figure shows the preoperative magnetic resonance imaging result of patient no. 1 in the HT group. (C2) Figure shows the postoperative magnetic resonance imaging result of patient no. 1 in the HT group. (D1) Figure shows the preoperative magnetic resonance imaging result of patient no. 2 in the HT group. (D2) Figure shows the postoperative magnetic resonance imaging result of patient no. 2 in the HT group.

We found significantly better reduction in thigh leg circumference with PLT grafts than HT grafts in ACLR. Agarwal et al also found significant improvement in thigh muscle atrophy in the PLT group called HT group at 1 year follow up after ACLR and these patients in the PLT group responded better to physiotherapy when recovering from thigh muscle atrophy.^[51]^ Dwidmuthe et al who observed changes in thigh leg circumference at different time points 2 weeks, 6 weeks, 3 months and 6 months after ACLR, showed a reduction in thigh circumference in the popliteal graft group as compared to the peroneus longus graft group, suggesting possible muscle atrophy in this group.^[54]^ In a randomized controlled study by Rhatomy et al, no patient in the PLT group showed thigh muscle atrophy of more than 20 mm, only 1 patient showed thigh muscle atrophy of 20 mm and 4 patients showed thigh muscle atrophy of 10 mm. At 1 year postoperatively, thigh muscle atrophy was significantly more severe in the popliteus tendon group, with a mean reduction in donor-area thigh circumference of 11.4 ± 3.6 mm in the popliteus group compared with the PLT group, which had a mean thigh circumference difference of 2.5 ± 0.5 mm.^[55]^ (We selected 2 patients from each of the PLT group and the HT group to compare the MRI results before and after their ACLR surgeries Fig. 2A1, A2, B1, B2, C1, C2, D1, D2.)

## 6. Conclusion

According to our retrospective study, There is no significant difference in postoperative knee function between autologous PLT and autologous HT in ACLR, but the thigh circumference of HT group is significantly reduced, while the state and function of the donor muscles around the ankle joint in PLT group are weakened. The 2 autogenous tendon grafts have their own advantages, and can be used in combination with the patient’s situation in clinical practice.

## Acknowledgments

Thanks for the guidance of Kaiwei Zhang, and the support of Guizhou University of Traditional Chinese Medicine and the First Affiliated Hospital of Guizhou University of Traditional Chinese Medicine.

## Author contributions

**Conceptualization:** Wenwen Zhang.

**Data curation:** Xu Gao, Yuhai Yan, Wenwen Zhang.

**Formal analysis:** Yuhai Yan.

**Methodology:** Kaiwei Zhang.

**Software:** Xu Gao, Yan Wang, Yuhai Yan, Wenwen Zhang.

**Supervision:** Kaiwei Zhang.

**Validation:** Ji Fei, Kaiwei Zhang.

**Visualization:** Ji Fei, Kaiwei Zhang.

**Writing – original draft:** Xu Gao, Yan Wang.

**Writing – review & editing:** Ji Fei, Kaiwei Zhang.
